# Sprint-interval but not continuous exercise increases PGC-1α protein content and p53 phosphorylation in nuclear fractions of human skeletal muscle

**DOI:** 10.1038/srep44227

**Published:** 2017-03-10

**Authors:** Cesare Granata, Rodrigo S. F. Oliveira, Jonathan P. Little, Kathrin Renner, David J. Bishop

**Affiliations:** 1 Institute of Sport, Exercise and Active Living (ISEAL), College of Sport and Exercise Science, Victoria University, Melbourne, Australia; 2 School of Health and Exercise Sciences, University of British Columbia Okanagan, Kelowna, British Columbia, Canada; 3 Department of Internal Medicine III, University Hospital of Regensburg, Regensburg, Germany

## Abstract

Sprint interval training has been reported to induce similar or greater mitochondrial adaptations to continuous training. However, there is limited knowledge about the effects of different exercise types on the early molecular events regulating mitochondrial biogenesis. Therefore, we compared the effects of continuous and sprint interval exercise on key regulatory proteins linked to mitochondrial biogenesis in subcellular fractions of human skeletal muscle. Nineteen men, performed either 24 min of moderate-intensity continuous cycling at 63% of W_Peak_ (CE), or 4 × 30-s “all-out” cycling sprints (SIE). Muscle samples (vastus lateralis) were collected pre-, immediately (+0 h) and 3 (+3 h) hours post-exercise. Nuclear p53 and PHF20 protein content increased at +0 h, with no difference between groups. Nuclear p53 phosphorylation and PGC-1α protein content increased at +0 h after SIE, but not CE. We demonstrate an exercise-induced increase in nuclear p53 protein content, an event that may relate to greater p53 stability - as also suggested by increased PHF20 protein content. Increased nuclear p53 phosphorylation and PGC-1α protein content immediately following SIE but not CE suggests these may represent important early molecular events in the exercise-induced response to exercise, and that SIE is a time-efficient and possibly superior option than CE to promote these adaptations.

Exercise training induces mitochondrial biogenesis^1^ and leads to an improved capacity for substrate oxidation, greater mitochondrial content, and/or increased mitochondrial respiration^2^,^3^,^4^,^5^,^6^. Exercise is therefore a valuable tool to help prevent and treat a host of chronic diseases linked with reduced and/or compromised mitochondrial function^7^. Current recommendations are that adults engage in moderate-intensity continuous exercise (CE) for ~30 min/d on 5 d/wk, or in vigorous-intensity exercise for ~20 min/d on 3 d/wk, or a combination of both^8^. Considering that lack of time is often cited as the primary reason for physical inactivity^9^, time efficient forms of exercise could improve exercise adherence and help prevent chronic diseases linked with a sedentary lifestyle^3^,^10^. One such type of exercise is low-volume sprint interval exercise (SIE), which consists of short “all-out” efforts interspersed with passive recovery^3^. Although sprint interval training has been shown to induce similar and sometimes greater mitochondrial and metabolic adaptations compared to continuous training^11^,^12^, there is limited information about the effects of SIE on the early molecular events regulating mitochondrial biogenesis, especially in comparison to longer duration CE.

A key mediator of exercise-induced mitochondrial biogenesis is peroxisome proliferator-activated receptor γ coactivator-1α (PGC-1α), which modulates gene transcription in the cellular nucleus^13^. In rat skeletal muscle, the increase in whole-muscle PGC-1α protein content following 3 hours of recovery is preceded by an increase in nuclear PGC-1α protein content immediately after the termination of exercise^14^. The authors suggested this increase in nuclear PGC-1α protein content may constitute the initial phase of the exercise-induced adaptive response. There is controversy however, regarding the timing and magnitude of the increase in nuclear PGC-1α protein content in human skeletal muscle following different types of exercise. While a single bout of SIE has been shown to induce an increase in nuclear PGC-1α protein content following 3 hours of recovery^15^, the effects of CE are controversial. One study has reported an increase in nuclear PGC-1α protein content immediately post-exercise^16^, whereas other studies observed no change either immediately post-exercise^17^ or after 3 hours of recovery^18^. These discrepancies may relate to the small sample size of the three studies employing CE (n = 4 to 6)^16^,^17^,^18^, and the fact that participants’ fitness levels and previous physical activity history were also different between studies. Moreover, all three of these studies investigated only a single time point, hence providing no indication of the timing of this change. Therefore, to better understand the influence of CE and SIE on the timing and magnitude of the change in nuclear PGC-1α protein content it is important to directly compare participants of similar fitness levels within the same study.

p53 is also a key transcription factor mediating exercise-induced mitochondrial biogenesis^19^, as demonstrated by its transcriptional control of mitochondrial respiration^20^ and mitochondrial remodelling^19^,^21^,^22^, as well as by the reduced mitochondrial content and exercise capacity in p53 knockout mice^20^,^23^. However, little is known about the effects of exercise on changes in p53 protein content in the nucleus, where p53 exerts its transcriptional and biochemical activity^24^. An increase in nuclear p53 protein content has been observed 3 hours after a 60-min bout of CE in human skeletal muscle^18^, whereas both an increase^25^ and a decrease^26^ have been reported following CE in rodent skeletal muscle; no study has investigated the effects of SIE. In addition to changes in content, phosphorylation of p53 at serine 15 (p-p53^Ser15^) is an important post-translational modification that enhances p53 activity and stability^27^. A single bout of CE increased p-p53^Ser15^ in human skeletal whole-muscle lysates^28^, whereas the effects of SIE remain unknown. Given that induction of the p53 response to physiological stressors, such as exercise, occurs largely through p53 protein stabilisation and nuclear accumulation^19^,^24^, it is important to investigate the exercise-induced changes in nuclear p-p53^Ser15^ in human skeletal muscle, and to determine whether these changes (and those in nuclear p53 protein content) may be differentially regulated by different types of exercise.

An important regulator of p53 is plant homeodomain finger-containing protein 20 (PHF20), which induces both p53 protein stability^29^ and p53 transcription^30^ in the nucleus. Research in human skeletal muscle has observed increased protein content of PHF20 (as well as p53 and PGC-1α) in whole muscle lysates following four weeks of “all-out” sprint training, but not moderate-intensity continuous training, and suggested exercise intensity may be associated with greater upregulation of both PGC-1α- and p53-mediated mitochondrial biogenesis^12^. Despite these findings, no research has investigated the impact of a single bout of exercise on nuclear PHF20 protein content in human skeletal muscle, and determined if the potential effects are differentially modulated by different types of exercise.

The purpose of this study was to compare the effects of a single bout of CE and SIE on key regulatory proteins associated with mitochondrial biogenesis in nuclear- and cytosolic-enriched fractions of human skeletal muscle. It was hypothesised that, similar to PGC-1α, exercise would increase the nuclear protein content of p53 and PHF20, and Ser-15 phosphorylation of p53 in the nucleus. It was also hypothesised that these increases would be greater following SIE, despite a shorter time commitment and a much lower total work.

## Results

### Total work, performance parameters and blood lactate concentration ([La^−^]) during the biopsy trial

Total work during the biopsy trial was higher for CE compared with SIE (3.6-fold, *P *<* *0.001; Table 1). In contrast, 1-s maximum and mean exercise intensity (expressed in Watts) were higher for SIE compared with CE (4.7- and 3.3-fold, respectively, all *P* < 0.001; Table 1). The exercise-induced increase in blood [La^−^] for the two types of exercise (both *P *<* *0.001) was also greater in SIE compared with CE (2.9-fold, *P *<* *0.001; Table 1).

### Muscle analyses

#### Representative immunoblots, cellular fractions enrichment, and antibody specificity

**Figure 1:**
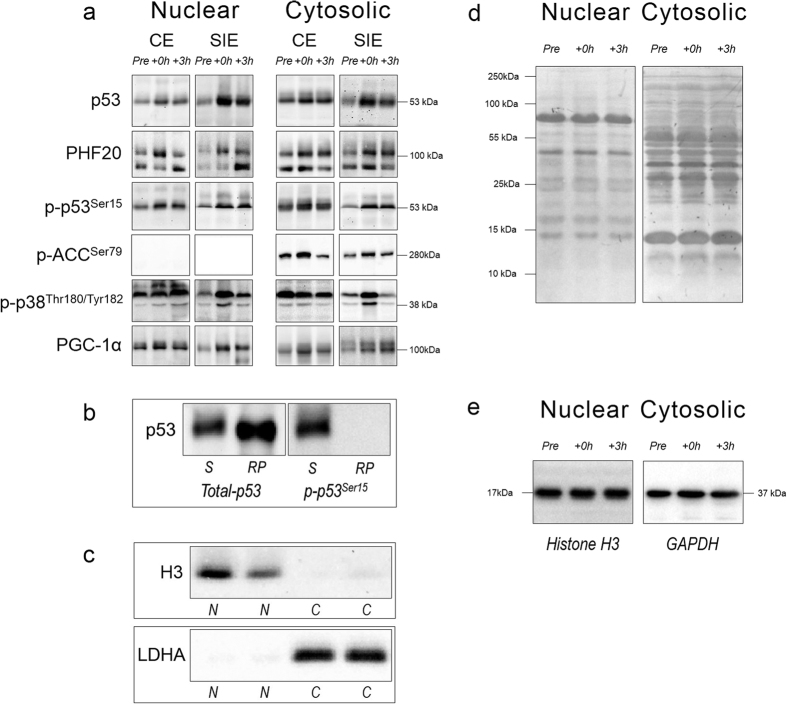
Representative immunoblots, p53 antibody specificity, subcellular enrichment and protein loading controls. (**a**) Representative immunoblots corresponding to total and phosphorylated protein content measured in the nuclear and cytosolic fractions, before (Pre), immediately (+0 h) and 3 h (+3 h) after the CE and SIE trials. p-p38 MAPK^Thr180/Tyr182^: bottom band at ~38 kDa; PHF20: top band at ~105 kDa. No band was detected in the nuclear fractions for p-ACC^Ser79^. (**b**) Confirmation of the p53 antibody specificity; samples were run beside an untagged full length p53 recombinant human protein and blotted against both total-p53 (positive control) and p-p53^Ser15^ (negative control) antibodies. S: sample; RP: p53 recombinant protein. (**c**) Histone H3 and LDHA were used as indicators of cytosolic and nuclear enrichment, respectively. N: nuclear fractions; C: cytosolic fractions. (**d**) Whole-lane Coomassie blue staining for both nuclear and cytosolic fractions, and (**e**) histone H3 (nuclear) and GAPDH (cytosolic), were used to verify equal loading between lanes. The immunoblot and whole-lane Coomassie images in this figure were cropped to improve the conciseness and clarity of the presentation.

Representative blots for the study are presented in Fig. 1a. The specificity of the p53 antibody was assessed by blotting samples beside a commercially-available, untagged, full-length p53 recombinant human protein (Aviva Systems Biology, AEP00002) (Fig. 1b). The same full-length p53 recombinant human protein, which was expressed in *E. coli* and was not phosphorylated, was also used as a negative control against the chosen p-p53^Ser15^ antibody. Results from Fig. 1b show that the p-p53^Ser15^ antibody did not recognise this protein, suggesting it is phospho-specific. The enrichment and purity of both nuclear and cytosolic fractions was confirmed by blotting the separated fractions against the nuclear protein histone H3, and the cytosolic protein lactate dehydrogenase A (LDHA). Histone H3 was only detected in nuclear fractions, whereas LDHA was only detected in cytosolic fraction (Fig. 1c), confirming the cellular fractionation protocol was successful. Images showing whole-lane Coomassie staining for both nuclear and cytosolic fractions, and histone H3 (nuclear) and GAPDH (cytosolic) immunoblots, were used to verify equal loading between lanes, and representative images are presented in Fig. 1d and e respectively.

#### p53 protein content

**Figure 2:**
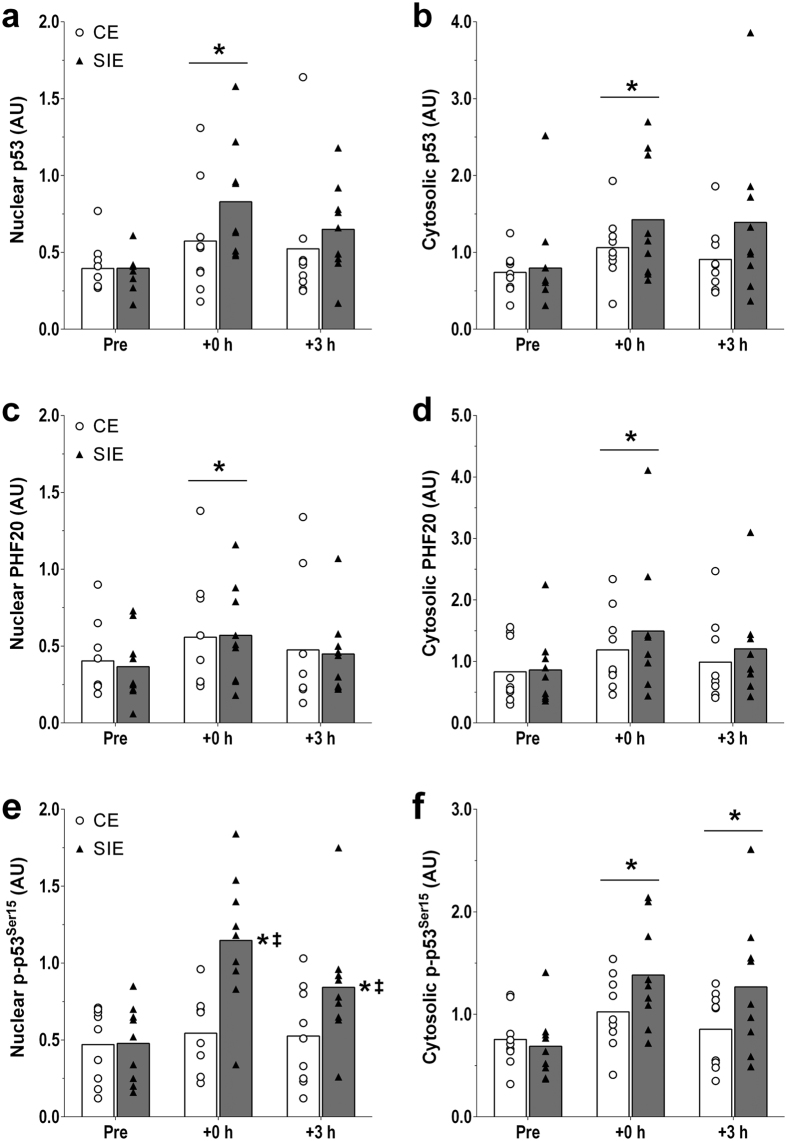
p53 and PHF20. Protein content of nuclear (**a**) and cytosolic (**b**) p53, of nuclear (**c**) and cytosolic (**d**) PHF20, and of nuclear (**e**) and cytosolic (**f**) p-p53^Ser15^ before (Pre), immediately (+0 h) and 3 h (+3 h) after the CE and SIE trials. Open circles (CE) and closed triangles (SIE) represent individual values. Primary effects were analysed using two-way ANOVA with repeated measures for time followed by Tukey’s honestly significant difference post-hoc test for pairwise comparisons. *P < 0.05 *vs*. Pre; ^‡^*P* < 0.05 *vs*. CE at the same time point. Individual data points and mean bars are plotted. n = 9 for each type of exercise.

There were main effects of time (nuclear: *P* = 0.002; cytosolic: *P* = 0.012), with the content of p53 protein significantly increased in both the nuclear (2.0-fold; *P* = 0.001) and cytosolic (1.8-fold; *P* = 0.014) fraction at +0 h only. There was no interaction effect (nuclear: *P* = 0.283; cytosolic: *P* = 0.393), indicating no significant difference in p53 protein content between the two types of exercise in either the nuclear (1.5-fold; ES: 1.61; −0.76, 3.97) or cytosolic (1.4-fold; ES: 0.57; −0.35, 1.48) fraction at +0 h. There were no significant differences versus baseline or between the two types of exercise at +3 h in either fraction. (Fig. 2a and b).

#### PHF20 protein content

There were main effects of time (nuclear: *P* = 0.006; cytosolic: *P* < 0.001), with the content of PHF20 protein significantly increased in both the nuclear (1.9-fold; *P* = 0.004) and cytosolic (1.7-fold; *P* < 0.001) fraction at +0 h only. There was no interaction effect (nuclear: *P* = 0.864; cytosolic: *P* = 0.399), indicating no significant difference in PHF20 protein content between the two types of exercise in either the nuclear (1.3-fold; ES: 0.22; −0.83, 1.26) or cytosolic (1.1-fold; ES: 0.49; −0.37, 1.34) fraction at +0 h. There were no significant differences versus baseline or between the two types of exercise at +3 h in either fraction. (Fig. 2c and d).

#### p-p53^Ser15^ protein content

There was an interaction effect (*P* = 0.003), with nuclear p-p53^Ser15^ content increased both at +0 h (3.1-fold; *P* < 0.001), and at +3 h (2.1-fold; *P* = 0.007), following SIE but not CE (1.5-fold, *P* = 0.785 at +0 h, and 1.2-fold, *P* = 0.873 at +3 h). p-p53^Ser15^ content was significantly greater in SIE compared with CE at both time points (2.1-fold; *P* < 0.001; ES: 2.40; 0.88, 3.43 at +0 h, and 1.8-fold; *P* = 0.044; ES: 1.25; −0.06, 2.55 at +3 h). There was a main effect of time in the cytosolic fraction (*P* < 0.001), with p-p53^Ser15^ content significantly increased at both time points (1.8-fold; *P* < 0.001 at +0 h, and 1.6-fold; *P* = 0.008 at +3 h). There was no interaction effect (*P* = 0.068), indicating no significant difference in p-p53^Ser15^ content between the two types of exercise at both time points (1.5-fold; ES: 1.38; −0.05, 2.81 at +0 h, and 1.7-fold; ES: 1.56; −0.25, 3.37 at +3 h).(Fig. 2e and f).

#### Phosphorylation of Acetyl-CoA Carboxylase (ACC) at serine 79 (p-ACC^Ser79^) protein content

**Figure 3:**
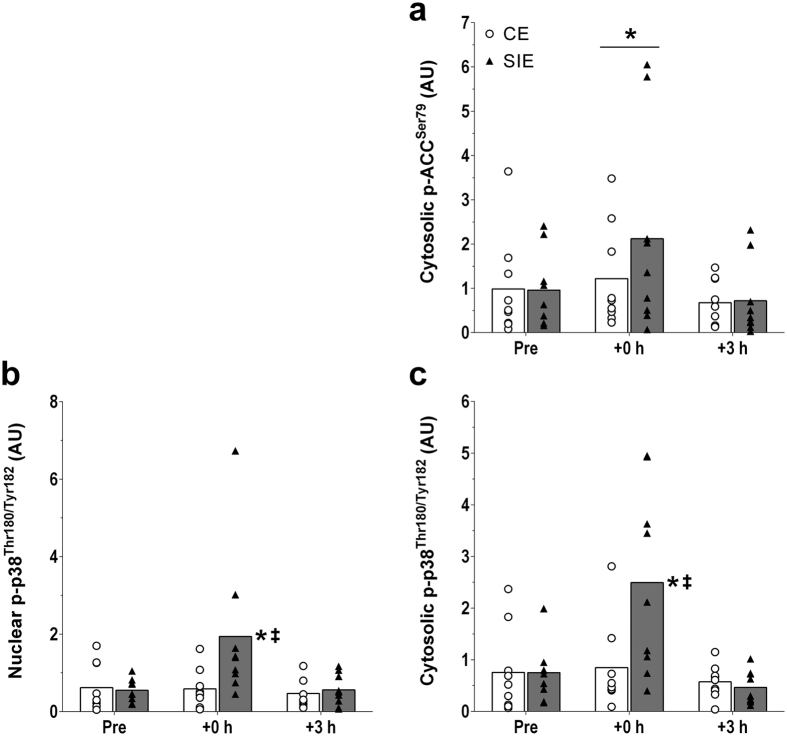
Signalling proteins. Protein content of cytosolic p-ACC^Ser79^ (**a**), and of nuclear (**b**) and cytosolic (**c**) p-p38 MAPK^Thr180/Tyr182^ before (Pre), immediately (+0 h) and 3 h (+3 h) after the CE and SIE trials. Open circles (CE) and closed triangles (SIE) represent individual values. Primary effects were analysed using two-way ANOVA with repeated measures for time followed by Tukey’s honestly significant difference post-hoc test for pairwise comparisons. *P < 0.05 *vs*. Pre; ^‡^*P* < 0.05 *vs*. CE at the same time point. Individual data points and mean bars are plotted. n = 9 for each type of exercise.

p-ACC^Ser79^ was not detected in nuclear fractions (Fig. 1a). There was a main effect of time in the cytosolic fraction (*P* < 0.001), with p-ACC^Ser79^ content significantly increased (1.7-fold; *P* = 0.012) at +0 h only. There was no interaction effect (*P* = 0.092), indicating no significant difference in p-ACC^Ser79^ content between the two types of exercise at +0 h (1.2-fold; ES: 0.92; −0.21, 2.05). There were no significant differences versus baseline or between the two types of exercise at +3 h (Fig. 3a).

#### Phosphorylation of p38 mitogen-activated protein kinase (MAPK) at threonine 180 and tyrosine 182 (p-p38 MAPK^Thr180/Tyr182^) protein content

There was an interaction effect (*P* = 0.035) in the nuclear fraction, with p-p38 MAPK^Thr180/Tyr182^ content increased 3.7-fold at +0 h following SIE (*P* = 0.004), but not CE (1.9-fold; *P* = 0.997). p-p38 MAPK^Thr180/Tyr182^ content was 2.0-fold greater in SIE compared with CE at this time point (*P* = 0.003; ES: 2.88; −0.17, 5.92). There was also an interaction effect (*P* = 0.003) in the cytosolic fraction, with p-p38 MAPK^Thr180/Tyr182^ content increased 3.9-fold at +0 h following SIE (*P* < 0.001), but not CE (1.9-fold; *P* = 0.966). p-p38 MAPK^Thr180/Tyr182^ content was 2.0-fold greater in SIE compared with CE at this time point (*P* < 0.001; ES: 2.34; 0.39, 4.29). There were no significant differences versus baseline or between the two types of exercise at +3 h in either fraction. (Fig. 3b and c).

#### Gene expression

**Figure 4:**
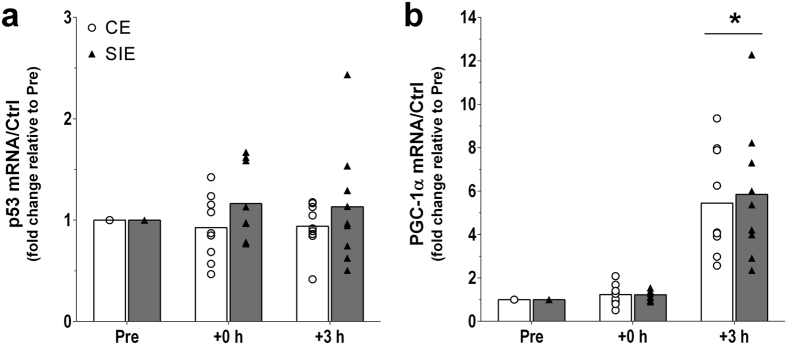
Gene expression. p53 (**a**), and PGC-1α (**b**) mRNA content before (Pre), immediately (+0 h), and 3 h (+3 h) after the CE and SIE trials, expressed relative to control (cyclophilin, glyceraldehyde 3-phosphate dehydrogenase [GAPDH], and beta-2-microglobulin [B2M] housekeeping genes). Open circles (CE) and closed triangles (SIE) represent individual values. Primary effects were analysed using two-way ANOVA with repeated measures for time followed by Tukey’s honestly significant difference post-hoc test for pairwise comparisons. *P < 0.05 *vs*. Pre. Individual data points and mean bars are plotted. n = 9 for each type of exercise.

There was no change in p53 mRNA content throughout (main effect of time: *P* = 0.883; Fig. 4a). There was a main effect of time for PGC-1α mRNA content (*P* < 0.001; Fig. 4b), with a significant increase (5.6-fold, *P* < 0.001) at +3 h only. There was no interaction effect (*P* = 0.910), indicating no significant difference in PGC-1α mRNA content between the two types of exercise at +3 h (1.1-fold; ES: 0.147; −0.85, 1.13); there were no significant differences versus baseline or between the two types of exercise at +0 h (all *P* > 0.05). Finally, results relative to the mRNA content of apoptosis-inducing factor (AIF), dynamin-related protein 1 (DRP1), mitofusin 2 (MFN2), p21, superoxide dismutase 2 (SOD2), and cytochrome *c* are presented in Table 2.

#### PGC-1α protein content

**Figure 5:**
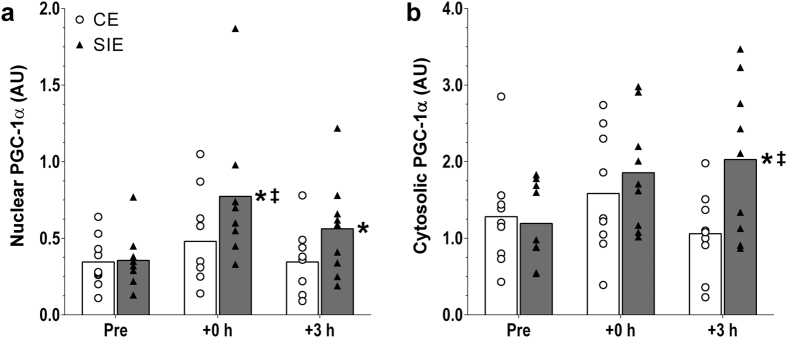
PGC-1α. Nuclear (**a**) and cytosolic (**b**) PGC-1α protein content before (Pre), immediately (+0 h) and 3 h (+3 h) after the CE and SIE trials. Open circles (CE) and closed triangles (SIE) represent individual values. Primary effects were analysed using two-way ANOVA with repeated measures for time followed by Tukey’s honestly significant difference post-hoc test for pairwise comparisons. *P < 0.05 *vs*. Pre; ^‡^*P* < 0.05 *vs*. CE at the same time point. Individual data points and mean bars are plotted. n = 9 for each type of exercise.

There was an interaction effect (*P* = 0.046), with nuclear PGC-1α protein content increased both at +0 h (2.3-fold; *P* < 0.001), and at +3 h (1.7-fold; *P* = 0.037), following SIE but not CE (1.4-fold; *P* = 0.219 at +0 h, and 1.0-fold; *P* = 1.000 at +3 h). PGC-1α protein content was significantly greater in SIE compared with CE at +0 h (1.7-fold; *P* = 0.043; ES: 1.61; 0.00, 3.22), but not at +3 h (1.6-fold; *P* = 0.130; ES: 1.17; 0.13, 2.21). There was an interaction effect (*P* = 0.036), with cytosolic PGC-1α protein content increased 1.9-fold at +3 h following SIE (*P* = 0.015), but not CE (1.1-fold; *P* = 0.702). PGC-1α protein content was significantly greater in SIE compared with CE at this time point (1.7-fold; *P* = 0.008; ES: 1.69; 0.21, 3.17). There were no significant differences versus baseline or between the two types of exercise at +0 h. (Fig. 5a and b).

## Discussion

This study reports, for the first time, that only SIE, and not CE, was associated with a post-exercise increase in p-p53^Ser15^ in the nuclear fraction of human skeletal muscle. Similarly, only SIE, and not CE, increased nuclear PGC-1α protein content post-exercise. Although the differential response to SIE and CE may be due to differences in the degree and time course of nuclear p53 and PGC-1α protein accumulation, our findings suggest that SIE may represent a more potent stimulus than CE to induce some of the early molecular events associated with mitochondrial biogenesis, as previously suggested^31^. An additional novel finding was that the exercise-induced increase in the nuclear content of p53 protein in human skeletal muscle was also accompanied by an increase in the nuclear content of PHF20, a protein that stabilises and activates p53^29^, but was not differentially regulated by the two types of exercise investigated.

Nuclear (and cytosolic) p53 protein content was increased immediately post-exercise, with no difference between the two types of exercise. Previous research in human skeletal muscle also reported an increase in nuclear p53 protein content after a 60-min bout of CE^18^; however, this was only reported after 3 hours of recovery (the only time point investigated in this previous study). Our results also agree with reports of increased nuclear p53 protein content immediately after a 60-min bout of continuous running in mouse skeletal muscle^25^, and 1 hour after a 20-min bout of intermittent eccentric skeletal muscle contractions in rats^32^. A decrease in nuclear p53 protein content has also been observed in one study after a 90-min bout of exhaustive exercise in mouse skeletal muscle^26^. This contrasting result is difficult to explain, but may relate to the type, age, and sex of the species investigated, and the different types of muscle analysed. We add the new finding that SIE also induced nuclear p53 protein accumulation, and that this increase was similar to that observed for CE. Our results are consistent with the well-accepted notion that cellular stress is associated with accumulation of p53 protein in the nucleus, a process that is mainly mediated by post-translational events such as nuclear/cytosolic shuttling, and/or increased p53 protein stability^24^,^33^.

p53 nuclear/cytosolic shuttling, a process requiring a tightly-regulated series of events^33^, cannot simply be assessed by subcellular fractionation and immunoblotting. We were therefore unable to determine the contribution of subcellular shuttling to the increase in nuclear p53 protein content observed with both types of exercise. However, some indirect measurements in the present study lend support to an exercise-induced increase in p53 stability. For example, and for the first time, we report a post-exercise increase in the nuclear content of PHF20, a multidomain protein that can bind to p53 and increase its stability^29^. In unstressed cells, nuclear p53 is bound to murine double minute-2 (MDM2), a p53 negative regulator inducing p53 ubiquitination and nuclear export, and subsequent cytosolic degradation by the proteasome^24^,^34^. Upon cellular stress PHF20 binds to p53, enhancing its stability by diminishing MDM2-mediated p53 degradation^29^. Although we were unable to directly measure the PHF20-p53 interaction, the concomitant increase in these two proteins may indicate an increased PHF20-p53 interaction induced by the cellular stress of exercise, and a subsequent increase in p53 stability^29^. Future research is required to confirm this hypothesis, and to better understand the molecular events regulating p53 stability, degradation, and nuclear/cytosolic shuttling following exercise.

An additional factor affecting p53 stability is phosphorylation at serine 15, which reduces the p53 interaction with its negative regulator MDM2^27^. In the present study, nuclear p-p53^Ser15^ was increased immediately post-exercise only following SIE, an increase that persisted until 3 h of recovery. Cytosolic p-p53^Ser15^ was also increased at both time points; however, the difference between the two types of exercise did not reach significance. While exercise duration and total work were greater during CE (1.7- and 3.6-fold, respectively), maximal and mean exercise intensity were 4.7-and 3.3- fold greater during SIE (Table 1). This suggests that exercise intensity, rather than exercise duration or total work, may be an important factor affecting exercise-induced changes in nuclear p-p53^Ser15^. The only two previous studies investigating p-p53^Ser15^ following endurance exercise with humans reported whole-muscle p-p53^Ser15^ to increase only after 3 h of recovery, regardless of exercise type (continuous or intermittent exercise at the same average intensity)^28^ or pre-exercise carbohydrate availability^35^. The earlier increase in nuclear p-p53^Ser15^ in our study (+0 h), compared with that in whole-muscle p-p53^Ser15^ in the two studies above (+3 h), raises the possibility that, similar to PGC-1α^14^, an increase in nuclear p-p53^Ser15^ may contribute to the initial phase of the exercise-induced adaptive response.

While multiple mechanisms probably contribute, the signalling kinases AMP-activated protein kinase (AMPK)^36^ and p38 MAPK^37^ have been reported to phosphorylate p53 at serine 15. In the present study, cytosolic phosphorylation of ACC, a downstream target and commonly used marker of AMPK activation^38^,^39^, was increased similarly between the two types of exercise immediately post-exercise. No ACC was detected in nuclear fractions, consistent with previous research^16^. Conversely, only SIE induced a significant increase in the phosphorylation of p38 MAPK in the nucleus, immediately post-exercise, consistent with the increase in nuclear p-p53^Ser15^ only following SIE. This may suggest that signalling through p38 MAPK may be more closely associated with the exercise-induced modulation of p-p53^Ser15^.

PHF20 can also act as a transcription factor, and has been shown to activate p53 gene expression^30^. Nonetheless, despite an increase in nuclear PHF20 protein content following both types of exercise, there was no significant change in p53 mRNA content within 3 h from the end of the bout. Studies in cells indicate that upregulation of p53 mRNA by PHF20 takes place only after 6 or 12 h^30^, suggesting that the 3 h post-exercise time point chosen in the present study might have been too early to detect a significant increase in p53 mRNA. Consistent with this, a small increase (~1.3-fold) has been reported following 3 h of recovery in human skeletal muscle with no difference between the three exercise intensities investigated^40^. Conversely, greater increases (2- to 2.5-fold) have been observed 4.5 and 7.5 h after the termination of the first running session of a twice a day exercise model^41^. The reasons for these divergent results may relate to the fact that participants engaged in eccentric exercise and that were fed before the post-exercise biopsies. Future research is required to clarify the controversial literature on this topic.

p53 is an inducible transcription factor that directly regulates the transcription of, amongst others, PGC-1α, DRP1, MFN2, AIF, p21 and SOD2 - a series of genes implicated in mitochondrial biogenesis^19^, mitochondrial remodelling^21^,^22^, cell survival^42^,^43^ and oxidative stress^44^. We report an exercise-induced increase in the mRNA content of these genes, although no significant differences between the two types of exercise were found. This is somewhat surprising considering the larger increase in nuclear p-p53^Ser15^ (and PGC-1α) after SIE compared with CE. Of note however, despite not reaching significance due to the large individual variability, the fold-change in p21 mRNA was almost twice as high following SIE compared to CE.

We observed an increase in nuclear PGC-1α protein content immediately after the termination of SIE (2.3-fold) and after 3 hours of recovery (1.7-fold). The increase we report after 3 h of recovery is similar to that observed in previous research in humans following an identical bout of SIE (1.7-fold)^15^; however, in the present study this increase was also observed immediately post-exercise. In contrast to SIE, CE (24 min at 63% of W_Peak_) did not provide a sufficient stimulus to induce a significant increase in nuclear PGC-1α protein content immediately post-exercise (1.4-fold), or after 3 hours of recovery (1.0-fold). Although these results may partially depend on the relatively short duration of the CE trial, they are consistent with the non-significant change (1.1-fold) in nuclear PGC-1α protein content reported immediately after 60 min of CE at 74% of 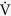
O_2Peak_^17^, the 1.5-fold increase observed immediately after a 90-min bout of CE at 65% of 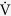
O_2Peak_^16^, and the non-significant change (0.70-fold) recorded 3 hours after a 60-min bout of CE at 70% of 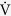
O_2Peak_^18^. Taken collectively, these results seem to indicate that CE at 60–75% of 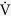
O_2Peak_ does not induce a large increase in nuclear PGC-1α protein content. However, by directly comparing participants of similar fitness levels within the same study, we report the novel finding that SIE is associated with larger increases in nuclear PGC-1α protein content than CE.

Similar to the exercise-induced increases in nuclear p-p53^Ser15^, and for the same reasons, the present results suggest that exercise intensity may also be an important factor affecting exercise-induced changes in nuclear PGC-1α protein content. Results from the cytosolic pool are also consistent with an exercise-intensity effect on the regulation of PGC-1α protein. Similar to the nuclear fraction, only SIE induced an increase in cytosolic PGC-1α protein content; however, this increase took place only after 3 h of recovery. The delayed increase in cytosolic compared with nuclear PGC-1α protein content provides further evidence that the initial phase of the exercise-induced adaptive response may indeed take place in the nucleus^14^.

The increase in the nuclear content of PGC-1α protein has previously been attributed to the exercise-induced translocation of PGC-1α from the cytosol to the nucleus^14^,^15^,^16^,^45^. While translocation remains possible, an alternative or additional explanation for the concomitant increase in nuclear and cytosolic PGC-1α protein content in the present study may relate to an increase in PGC-1α stability. An increase in p38 MAPK activity has previously been reported immediately post-exercise in humans^15^,^16^,^17^,^28^, and has been linked with greater PGC-1α stability^46^. The greater increase in the phosphorylation of p38 MAPK following SIE compared with CE in both subcellular fractions (both by 2.0-fold) occurred concomitantly with a greater increase in both nuclear and cytosolic PGC-1α protein content (both by 1.7-fold). It is therefore possible that exercise intensity may modulate PGC-1α protein content via an increase in PGC-1α stability, which may be mediated, at least in part, by greater phosphorylation of p38 MAPK^46^.

The PGC-1α protein has been shown to activate its own promoter through a feed-forward loop^47^; as a result, increased content of nuclear PGC-1α protein should further enhance PGC-1α transcriptional activity before degradation^45^. However, despite a greater increase in nuclear PGC-1α protein content following SIE, when compared to CE, there was no difference for the increase in PGC-1α mRNA content following the two exercise types. This highlights a potential dissociation between exercise-induced increases in PGC-1α nuclear protein content and PGC-1α mRNA (and the mRNA of cytochrome *c*, a downstream target of PGC-1α^48^). This may be related to other factors that influence PGC-1α transcriptional activity such as AMPK, a signalling protein inducing PGC-1α activation^39^,^49^, and its downstream target and commonly used biomarker ACC^38^,^39^, the phosphorylation of which was similarly increased following both exercise types. The similar increase in PGC-1α mRNA between the two types of exercise is also consistent with previous research comparing exercise at intensities below and above W_Peak_^40^,^50^, and is in agreement with the notion that the reported exercise intensity-dependent regulation of PGC-1α mRNA^51^ is limited to submaximal (i.e., <W_Peak_) exercise intensities^40^.

Although the between-subject design represents a potential limitation of this study, our findings add new insight into the early molecular events that regulate skeletal muscle remodelling in response to a single bout of exercise, and the role of exercise intensity in mediating these events. We report that a single bout of exercise induces nuclear accumulation of p53 protein, an increase that may relate to greater p53 stability, as suggested by the concomitant increase in PHF20 protein content. In addition, nuclear p-p53^Ser15^, a post-translational event also associated with enhanced p53 stability, and nuclear PGC-1α protein content, increased only following SIE, suggesting that exercise intensity may play an important role in the exercise-induced adaptations mediated by both p53 and PGC-1α. Results from the present study also indicate that increases in nuclear p-p53^Ser15^, as well as nuclear PGC-1α protein content, may represent important early events in the adaptive response to exercise. Our findings indicate that “all-out” SIE represents a valuable and possibly superior option to moderate-intensity CE for promoting the early molecular events leading to exercise-induced mitochondrial biogenesis, in a time-efficient manner.

## Methods

### Participants

Twenty healthy men aged 18–35 years, who were non-smokers, free of medications, moderately-trained (i.e., engaging in less than 3–4 hours per week of moderate, unstructured aerobic activity for 6 months prior to the study), and not regularly engaged in cycling-based sports, volunteered to participate in this research. Following medical screening participants were informed of the study requirements, benefits, and risks, before giving written informed consent. Approval for all the experimental protocols and the study’s procedures, which conformed to the standards set by the latest revision of the Declaration of Helsinki, was granted by the Victoria University Human Research Ethics Committee. All experiments and procedures were performed in accordance with the relevant guidelines and regulations set by the above Human Research Ethics Committee.

### Study design and testing

The experimental protocol consisted of two tests - a graded exercise test (GXT), and an exercise/biopsy trial. Participants were familiarised with both tests (with the exclusion of muscle biopsies) and were required to refrain from any strenuous exercise for the 72 h preceding each test, from alcohol and any exercise for 24 h before testing, and from food and caffeine consumption for 3 h before each test. After baseline testing, participants were ranked based on their W_LT_ and assigned in reversed counterbalanced order (ABBA) to the CE or SIE group (both, n = 10), in a between-subjects study design. The between-subject design was necessary as this study was part of a longer training study in which participants then repeated their assigned exercise trial for four weeks, as previously described^12^. Nineteen participants completed the study, with one participant (SIE group) withdrawing due to time constraints. Participants’ baseline physiological parameters are described in Table 1.

#### GXT

A discontinuous graded exercise test was performed on an electronically-braked cycle ergometer (Lode Excalibur, v2.0, The Netherlands) to determine the HR_Peak_, 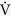
O_2Peak_, W_Peak_, W_LT_ (using the modified D_Max_ method^52^), and the exercise intensity for the biopsy trial, as previously described^12^ (Table 1). The W_Peak_ was determined as the power of the last completed stage plus 7.5 W for every additional minute completed. Expired air was continuously analysed for O_2_ and CO_2_ concentrations via a pre-calibrated gas analyser (Moxus 2010, AEI technologies, USA), and 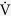
O_2_ values were recorded every 15 s. The average of the two highest consecutive 15-s values was recorded as a participant’s 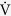
O_2Peak_. Glass capillary tubes were used to collect ~50 μL of blood prior to the test, and after each 4-min stage. Capillary blood [La^−^] was determined using a pre-calibrated blood-lactate analyser (YSI 2300 STAT Plus, YSI, USA).

#### Biopsy trial

All trials were performed in the morning to avoid variations caused by circadian rhythms. To minimise variability in muscle gene and protein expression attributable to diet, participants were provided with a standardised dinner (55 kJ·kg^−1^ body mass (BM), providing 2.1 g carbohydrate·kg^−1^ BM, 0.3 g fat·kg^−1^ BM, and 0.6 g protein·kg^−1^ BM) and breakfast (41 kJ·kg^−1^ BM, providing 1.8 g carbohydrate·kg^−1^ BM, 0.2 g fat·kg^−1^ BM, and 0.3 g protein·kg^−1^ BM), to be consumed 15 and 3 h prior to the biopsy trials, respectively. While resting in the supine position, and after injection of a local anaesthetic (1% xylocaine) into the skin and fascia of the vastus lateralis muscle, three small incisions were made about 2–3 cm apart. A resting muscle biopsy (Pre) was obtained using a biopsy needle with suction before participants began either the CE or SIE protocol an electronically-braked cycle ergometer (Velotron, RacerMate, USA). A second muscle biopsy (+0 h) was obtained immediately upon completion of the exercise bout (<5 s). A third muscle biopsy (+3 h) was obtained after 3 hours of recovery in the supine position (with no access to food and access to water *ab libitum*). Once obtained, muscle samples were immediately cleaned of excess blood, fat, and connective tissue, were rapidly frozen in liquid nitrogen, and stored at −80 °C for subsequent analyses. Capillary blood samples to measure [La^−^] were collected at rest, and immediately after the completion of exercise.

#### CE trial

Exercise consisted of 24 min of continuous cycling at a fixed power equivalent to 90% of W_LT_. This was preceded by a warm-up involving cycling for 6 min at 66% of W_LT_ followed by 2 min of rest. Exercise intensity was set relative to W_LT_ rather than W_Peak_ as metabolic and cardiac stresses are similar when individuals of differing fitness levels exercise at a percent of the W_LT_, but can vary significantly when exercising at a percent of W_Peak_^53^. Participants received consistent verbal encouragement for the duration of the exercise bout. The overall duration of the CE exercise protocol (32 min of exercise inclusive of warm-up) was chosen based on the physical activity guidelines set by the ACSM position stand^8^.

#### SIE trial

Following the same warm-up procedure for CE, SIE consisted of 4 × 30-s “all-out” cycling bouts against a resistance set at 0.075 kg·kg^−1^ BM, interspersed with 4 min of rest (2 min of total exercise; 22 min of total session duration inclusive of warm-up). Participants received consistent verbal encouragement to keep the cadence as high as possible during the entire duration of the bout.

### Skeletal muscle analyses

#### Subcellular fractionation

Nuclear and cytosolic fractions were prepared from 40–60 mg of wet muscle using a commercially-available nuclear extraction kit (NE-PER, Pierce, USA). Muscle samples were homogenised in CER-I buffer containing a protease/phosphatase inhibitor cocktail (Cell Signaling Technology [CST], 5872). Following centrifugation the supernatant was taken as the crude cytosolic fraction. Pellets containing nuclei were washed five times in PBS to remove cytosolic contamination, before nuclear proteins were extracted by centrifugation in high-salt NER buffer supplemented with the same inhibitors cocktail following manufacturers’ instruction. Sufficient muscle was available to prepare subcellular fractions from nine participants in each group. Verification of subcellular enrichment is presented in the Results section.

#### Immunoblotting

Protein concentration was determined in triplicate using a commercial colorimetric assay (Bio-Rad Protein Assay kit-II, Australia). Muscle lysates (10–50 μg) were separated by electrophoresis using SDS-PAGE gels (8–15%) as previously described^12^. The following primary antibodies were used (supplier, catalogue number): histone H3 (CST, 9715), LDHA (CST, 2012), p53 (CST, 2527), PGC-1α (Calbiochem, st-1202), p-ACC^Ser79^ (CST, 3661), p-p38 MAPK^Thr180/Tyr182^ (CST, 9211), p-p53^Ser15^ (CST, 9284), and PHF20 (CST, 3934). An internal standard was loaded in each gel, and each lane was normalised to this value, to reduce gel-to-gel variability. Whole-lane Coomassie blue staining, and immunoblots for H3 (nuclear) and GAPDH (cytosolic) were performed to verify correct loading and equal transfer between lanes^54^. Representative blots and loading control images are presented in Fig. 1.

#### Total RNA isolation

Total RNA was isolated from approximately 15–25 mg of muscle tissue using the RNeasy^®^ Mini kit (Qiagen, Canada) according to the manufacturer’s instructions. Muscle samples were homogenised using the TissueLyser II (Qiagen, Canada), and total RNA was isolated from the aqueous phase following precipitation with 600 μL of 70% ethanol using RNeasy^®^ Mini kit. On-column DNA digestion was performed. RNA concentration was determined by spectrophotometry (Nanodrop ND1000, Thermo Fisher Scientific, USA) by measuring the absorbance at 260 nm (A260) and 280 nm (A280), with A260/A280 ratios above 1.8 indicating high-quality RNA. Sufficient muscle was available to isolate and analyse total RNA from nine participants in each group.

#### Real-time RT-PCR

First-strand cDNA synthesis from 500 ng of total RNA was performed with random hexamer primers using a high-capacity cDNA reverse transcription kit (Applied Biosystems, USA), according to manufacturer’s directions. All samples and reverse transcriptase (RT) negative controls were run together to prevent technical variation. Forward and reverse primers for the target and housekeeping genes (Table 3) were designed based on NCBI RefSeq using NCBI Primer-BLAST (www.ncbi.nlm.nih.gov/BLAST/). Specificity of the amplified product was confirmed by melting point dissociation curves. The mRNA expression of AIF, cyt *c*, DRP1, MFN2, p21, p53, PGC-1α and SOD2 were quantified by quantitative real-time RT-PCR (Mastercycler^®^ RealPlex2, Eppendorf, Germany), using a 10 μL PCR reaction volume and SYBR Green chemistry (iTaqTM Universal SYBR^®^ Green Supermix, Bio-Rad, USA). All samples were run in duplicate simultaneously with template free controls, using an automated pipetting system (epMotion 5070, Eppendorf, Germany). The following PCR cycling patterns were used: initial denaturation at 95 °C (3 min), 40 cycles of 95 °C (15 s) and 60 °C (60 s). Relative changes in mRNA content were calculated using the normalised relative quantities (NRQs) method^55^. To account for the efficiency of RT and initial RNA concentration, the mRNA expression of four housekeeping genes was quantified, and their stability was determined using the BestKeeper software^56^. Cyclophilin, glyceraldehyde 3-phosphate dehydrogenase (GAPDH), and beta-2-microglobulin (B2M) were classified as stable, whereas TATA-binding protein (TBP) was reported as unstable and was therefore excluded. These results were confirmed by the Normfinder algorithm^57^.

### Statistical analysis

All values are reported as mean ± SD unless otherwise specified. Unpaired t-tests were used to assess differences between SIE and CE for Pre values in immunoblot analyses, and for age, height, body mass, HR_Peak_, W_LT_, W_Peak_, 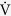
O_2Peak_, as well as 1-s max and mean exercise intensity, and total work during the biopsy trial. To investigate the influence of exercise type and time, and the interaction between both of these variables, two-way ANOVA with repeated measures for time were used. Where no interaction effects were observed, pooled values for time are reported. Significant interactions and main effects were further analysed using Tukey’s honestly significant difference post-hoc test. Sigma Stat software (Jandel Scientific, USA) was used for all statistical analyses. The level of statistical significance was set a priori at *P* < 0.05. To assess the magnitude of effects, effect sizes (ES), assessed using Cohen’s *d*, and 95% confidence intervals (95% CI), were also calculated and are reported as (ES; 95% CI) of the between-group difference (CE *vs*. SIE) scores.

## Additional Information

**How to cite this article**: Granata, C. *et al*. Sprint-interval but not continuous exercise increases PGC-1α protein content and p53 phosphorylation in nuclear fractions of human skeletal muscle. *Sci. Rep.* **7**, 44227; doi: 10.1038/srep44227 (2017).

**Publisher's note:** Springer Nature remains neutral with regard to jurisdictional claims in published maps and institutional affiliations.

## Figures and Tables

**Table 1:** Baseline characteristics of the participants; total work, performance parameters, and blood [La^−^] during the CE and SIE trials.

Measurement		CE (n = 10)	SIE (n = 9)
Age (y)		21 ± 2	21 ± 3
Body mass (kg)		79.8 ± 12.7	84.5 ± 19.4
Height (cm)		179.6 ± 6.1	180.6 ± 7.3
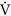 O_2Peak_ (mL min^−1^ kg^−1^)		47.0 ± 3.7	47.1 ± 7.8
W_LT_ (W)		194.8 ± 43.4	204.4 ± 39.7
W_Peak_ (W)		276.5 ± 51.5	280.8 ± 48.2
Total work (kJ)		252.6 ± 59.7	69.5 ± 9.1^‡^
1-s max exercise intensity (W)		175.4 ± 39.1	823.4 ± 153.5^‡^
1-s max exercise intensity (%W_Peak_)		63.1 ± 3.1	294.3 ± 33.7^‡^
Mean exercise intensity (W)		175.4 ± 39.1	578.8 ± 75.6^‡^
Mean exercise intensity (%W_Peak_)		63.1 ± 3.1	207.7 ± 14.0^‡^
Mean exercise intensity (%HR_Peak_)		81.3 ± 3.6	n/a
Max exercise intensity (%HR_Peak_)		n/a	92.8 ± 6.7
Blood [La^−^] (mmol L^−1^)	Pre	0.9 ± 0.2	0.9 ± 0.2
	+0 h	3.9 ± 0.3*	11.6 ± 1.4*^‡^

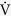
O_2Peak_: peak oxygen uptake; W_LT_: power at the lactate threshold; W_Peak_: peak power output; HR_Peak_: peak heart rate. Unpaired t-tests were used to assess differences between SIE and CE for age, body mass, height, HR_Peak_, 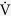
O_2Peak_, W_LT_, W_Peak_ at baseline, as well as total work, and 1-s max and mean exercise intensity during the biopsy trial. Changes in blood [La^−^] were analysed using a two-way ANOVA with repeated measures for time followed by Tukey’s honestly significant difference post-hoc test for pairwise comparisons. ^‡^*P* < 0.05 *vs*. CE at the same time point; *P < 0.05 *vs. P*re. All values are mean ± SD. n = 9 for each type of exercise.

**Table 2:** Fold-change relative to Pre, measured immediately (+0 h) and 3 h (+3 h) following the CE and SIE trials, for the mRNA content of AIF, DRP1, MFN2, p21, SOD2, and cyt *c*.

Gene	Time Point	CE (n = 9)	SIE (n = 9)
AIF	+0 h*	1.31×/÷0.52	1.53×/÷1.04
	+3 h	1.16×/÷0.39	1.47×/÷1.14
DRP1	+0 h*	1.21×/÷0.41	1.26×/÷0.32
	+3 h	0.98×/÷0.25	1.12×/÷0.50
MFN2	+0 h*	1.45×/÷0.65	1.43×/÷0.66
	+3 h	1.23×/÷0.48	1.12×/÷0.82
p21	+0 h	4.15×/÷5.06	4.48×/÷2.80
	+3 h*	13.07×/÷19.46	24.87×/÷29.77
SOD2	+0 h*	1.14×/÷0.37	1.27×/÷0.20
	+3 h*	1.16×/÷0.26	1.20×/÷0.19
cyt *c*	+0 h*	1.17×/÷0.17	1.17×/÷0.28
	+3 h*	1.24×/÷0.19	1.10×/÷0.25

Values are expressed relative to cyclophilin, GAPDH and B2M housekeeping genes.

Primary effects were analysed using two-way ANOVA with repeated measures for time followed by Tukey’s honestly significant difference post-hoc test for pairwise comparisons. *P < 0.05 *vs*. Pre. All values are mean×/÷SD. n = 9 for each type of exercise.

**Table 3:** Primers used for real-time RT-PCR analyses of mRNA expression.

Gene	Forward primer (5′ → 3′)	Reverse primer (5′ → 3′)
AIF	GATTGCAACAGGAGGTACTCCAAGA	GATTTGACTTCCCGTGAAATCTTCTC
cyt *c*	GGGCCAAATCTCCATGGTCT	TCTCCCCAGATGATGCCTTT
DRP1	CACCCGGAGACCTCTCATTC	CCCCATTCTTCTGCTTCCAC
MFN2	CCCCCTTGTCTTTATGCTGATGTT	TTTTGGGAGAGGTGTTGCTTATTTC
p53	GTTCCGAGAGCTGAATGAGG	TTATGGCGGGAGGTAGACTG
PGC-1α	GGCAGAAGGCAATTGAAGAG	TCAAAACGGTCCCTCAGTTC
p21	GCAGACCAGCATGACAGATTT	GATGTAGAGCGGGCCTTTGA
SOD2	CTGGACAAACCTCAGCCCTA	TGATGGCTTCCAGCAACTC
cyclophilin	GTCAACCCCACCGTGTTCTTC	TTTCTGCTGTCTTTGGGACCTTG
GAPDH	AATCCCATCACCATCTTCCA	TGGACTCCACGACGTACTCA
B2M	TGCTGTCTCCATGTTTGATGTATCT	TCTCTGCTCCCCACCTCTAAGT
